# Open questions: Tackling Darwin’s “instincts”: the genetic basis of behavioral evolution

**DOI:** 10.1186/s12915-017-0369-3

**Published:** 2017-04-03

**Authors:** J. Roman Arguello, Richard Benton

**Affiliations:** Center for Integrative Genomics, Génopode Building, Faculty of Biology and Medicine, University of Lausanne, CH-1015 Lausanne, Switzerland

## Abstract

All of us have marveled at the remarkable diversity of animal behaviors in nature.

None of us has much idea of how these have evolved.

## ᅟ

**Fig. 1.**
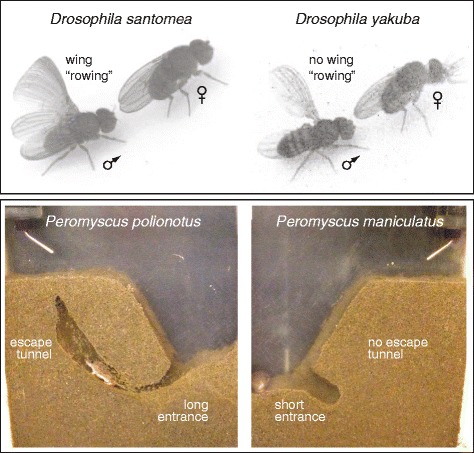
Evolving behaviors. Examples of genetically defined behaviors that differ between closely related species. *Top*: Differences in the use of wing “rowing” in the male courtship routine of drosophilid species, illustrated by chronophotography [17]. *Bottom*: Differences in burrow construction between *Peromyscus* deer mice species [10] (image credit: Brant Peterson and Hopi Hoekstra)

Like other inherited phenotypes, many behavioral traits of animals—predatory instincts, courtship rituals, and shelter building, to name but a few—have a genetic basis (Fig. 1). Genes, of course, don’t control behavior directly, but encode the vast array of molecules that establish the connectivity and physiology of the nervous system (to make no mention of those that form the tissues and organs in which neural circuits are embedded). What is the genetic basis by which seemingly complex behaviors have evolved?

This grand question covers a multitude of issues. Is there a subset of genes that have “special” roles in dictating behavioral evolution, or does behavior evolve along many different trajectories? What is the relative contribution of structural and regulatory genetic changes (influencing protein function and gene expression, respectively) to this process? What part(s) of the nervous system do such genetic changes impact: sensory input channels, central processing circuit and/or in locomotor pathways that directly control actions? Do answers to these questions vary among taxa?

### Why are these questions timely?

Technical and intellectual reasons. We’re getting very good at observing and quantifying behavior. Cameras and computers do much of the job, revealing behaviors that previously escaped detection by the human eye or which would be too laborious to measure manually [1]. With large-scale population surveys and comparative genomics, we’re also more attuned to the intra- and interspecific behavioral [2] and genetic variation [3] that exists around the world. We also simply now know a lot about the genetic and neural basis of behavior of well-established genetic model systems, such as the mouse *Mus musculus*, the fly *Drosophila melanogaster* and the worm *Caenorhabditis elegans*. These models provide valuable points of comparison with related species that display obvious (and presumably evolutionarily significant) variants in their behaviors. Importantly, with new genome editing methods (such as CRISPR/Cas9 [4]) and optogenetic and thermogenetic tools for turning on and off neurons at will [5], we’re potentially able to manipulate molecules and circuits with precision in such non-traditional model species. This ability will allow us to determine causal relationships—and not simply correlations—between genetic and behavioral variation.

### Why should neurobiologists care about these questions?

Despite impressive advances, we’re still a long way from understanding the genetic and neural basis of even simple behaviors. Seymour Benzer’s seminal work in the 1960s with *D. melanogaster* showed how forward genetics can identify key components of specific behaviors, such as courtship or circadian activity [6]. Despite the doors this work opened, the opposing view of Benzer’s contemporary, Jerry Hirsch, that behaviors are too complex to be reducible to the action of single (or a few) genes, is of course largely true. In flies and worms, large-scale genetic screens for loci underlying, for example, embryonic segmentation or axon guidance have been extraordinarily fruitful [7]. By contrast, behavioral screens are much harder to perform and, because there are many uninteresting ways in which a particular behavior can be disrupted, truly informative mutations are likely to be rare. Indeed, most characterized examples affect genes that have non-pleiotropic functions in peripheral sensory systems, such as specific olfactory receptors [2, 8]. Thus, a comparative, evolutionary approach that takes advantage of naturally occurring, phenotypically consequent genetic variants can offer a complementary way to identify molecular determinants of behavior. These may open new doors into explorations of the underlying neural circuits.

### Why should evolutionary biologists care about these questions?

Although behavioral traits are critical to animals’ survival and reproduction, the challenge of high-throughput and robust quantification of these often-complex phenotypes has hampered exploration of their evolution, especially by comparison with studies of, for example, morphological characteristics [9]. This is changing: the current intersection of phenotyping and genomic technologies is rapidly increasing our ability to link behavioral variation with specific regions of the genome, through quantitative trait locus and association mapping [2]. Importantly, these advances not only allow determination of the genetic architecture of behavioral variations (as in burrow building by *Peromyscus* mice; Fig. 1) [10]), but can also allow us to nail the causal gene(s) and genetic variant(s), as exemplified by studies of drosophilid courtship song [11] and *C. elegans*’ sensitivity to environmental gases [12, 13].

These tools can also enrich our ability to understand the evolutionary processes that govern behavioral divergence. Reverse genetic approaches, in which regions of the genome are identified because they carry signatures of selection, are often a starting point for this endeavor [14]. While the behavioral impact of such candidate regions has rarely been characterized experimentally, deepened knowledge of nervous systems and technical advances now make hypothesis generation and testing much more easily achieved. The increased ability to integrate evolutionary processes (e.g., mutation, selection, genetic drift) with neurobiological mechanisms will translate into a richer understanding of behavioral divergence. This, in turn, may help us understand to what extent selective versus non-selective forces are responsible for the evolution of behavior.

### What questions (and challenges) are there in the long-term?

Most initial traction on the problem of behavioral evolution is likely to come from studies of simple innate actions that can be reproduced in the laboratory. However, once efficient assays are established, we can begin to tackle behaviors that are shaped by internal state and experience, in essence trying to understand the evolutionary plasticity of neural plasticity. What remains a looming challenge is to place our favorite behavioral differences within an ecological context and understand the impact of these differences on fitness. This will be difficult for many reasons: fitness effects may be extremely small (though still impactful on an evolutionary scale) and many of our current model species’ behaviors in nature are poorly appreciated. Insights may thus only come from large-scale, field-based studies. Serious consideration should also be given to concerted efforts to establish new model species [15] which retain a close relationship with current model systems but have better-understood ecologies.

In his landmark book [16], Darwin opened his chapter on “Instinct” with a characteristically cautious statement: “*I have nothing to do with the origin of the primary mental powers, any more than I have with that of life itself*”. Over 150 years later, we might now be cautiously optimistic that an understanding of the evolution of at least some “mental powers” is within our reach.
